# Interfering histone deacetylase 4 inhibits the proliferation of vascular smooth muscle cells via regulating MEG3/miR-125a-5p/IRF1

**DOI:** 10.1080/19336918.2018.1506653

**Published:** 2018-08-29

**Authors:** Xiangtao Zheng, Ziheng Wu, Ke Xu, Yihui Qiu, Xiang Su, Zhen Zhang, Mengtao Zhou

**Affiliations:** a Department of Vascular Surgery, The First Affiliated Hospital of Wenzhou Medical University, Wenzhou, China; b Department of Vascular Surgery, The First Affiliated Hospital, School of Medicine, Zhejiang University, Hangzhou, China; c Department of Surgery, The First Affiliated Hospital of Wenzhou Medical University, Wenzhou, China

**Keywords:** VSMCs, HDAC4, MEG3, miR-125a-5p, IRF1

## Abstract

In this study, we investigated the role ofhistone deacetylase 4 (HDAC4) and MEG3/miR-125a-5p/interferonregulatoryfactor 1 (IRF1) on vascular smooth muscle cell (VSMCs)proliferation. Platelet derived growth factor (PDGF)-BB was used toinduce the proliferation and migration of VSMCs. The expressionsof MEG3, miR-125a-5p, HDAC4 and IRF1in VSMCs were detectedby qRT-PCR and western blot, respectively. ChIP assay was usedto determine the relationship between MEG3 and HDAC4. Doubleluciferase reporter assay was used to test the regulation betweenmiR-125-5p and IRF1. Results showed that PDGF-BB decreasedthe expression of MEG3 and IRF1, while increased the expressionof miR-125a-5p and HDAC4. In addition, HDAC4 knockdowninhibited the proliferation and migration of VSMCs via upregulatingMEG3 and downregulating miR-125a-5p. MiR-125a-5p inhibitorcould repress the proliferation and migration of VSMCs andalleviate intimal hyperplasia (IH) by directly upregulating IRF1expression. These results suggested that HDAC4 interferenceinhibited PDGF-BB-induced VSMCs proliferation via regulatingMEG3/miR-125a-5p/IRF1 axis, and then alleviated IH.

## Introduction

Vascular smooth muscle cells (VSMCs) are the main components of the vascular wall. Its abnormal proliferation is the major pathological basis for vascular proliferative disorders, such as atherosclerosis, and postangioplasty stenosis, which plays an important role in intimal hyperplasia (IH) [1]. Under normal conditions, VSMCs are in a quiescent state of growth, while vascular injury can lead to their uncontrolled proliferation [2]. Therefore, inhibiting the abnormal proliferation of VSMCs could play a role in alleviating IH.

Histone deacetylase 4 (HDAC4) belongs to the class Ⅱ histone acetylation enzyme (histone deacetylases, HDACs), and it demonstrates deacetylase activity. In recent years, it has been reported that HDAC4 can play an important role in the development of various diseases by participating in biological processes, such as the regulation of gene transcription and cell proliferation [3–5]. Ginnan et al. [6] found that overexpression of HDAC4 could promote the proliferation of VSMCs, which suggested that VSMCs abnormal proliferation might be inhibited by interfering with the expression of HDAC4. However, the mechanism of HDAC4 on VSMCs proliferation is not clear.

Long non-coding RNA (lncRNA) is a class of RNA with the length that greater than 200 nt. LncRNAs participate in a variety of biological processes, including cell apoptosis, proliferation, angiogenesis, immune responses, and protein modification. The lncRNA maternally expressed gene 3 (lncRNA MEG3), a tumor suppressor, has been shown to be associated with the proliferation, migration, and invasion of many cancers, like colorectal cancer, gastric cancer, prostate cancer [7]. Recent studies have indicated that MEG3 is related to the proliferation of VSMCs, and overexpression of MEG3 inhibits VSMCs proliferation [8,9]. However, the regulation mechanism has not been fully elucidated.

Interferon-regulatory factor 1 (IRF1), a transcription factor, was discovered in the 1988 by Miyamoto, which could combine the upstream of the IFN-β and regulate its expression [10]. It has been demonstrated that IRF1 is related to immune, tumor, and other related diseases, and can participate in the regulation of cell cycle and apoptosis. In the study of VSMCs, Zhang et al. [11] revealed that overexpression of IRF1 could inhibit cell-cycle progression in VSMCs via downregulating the expression of cyclin D1 and cyclin dependent kinase 4, suggesting an important role of IRF1 in VSMCs proliferation. Additionally, bioinformatics analysis (microRNA.org) found that IRF1 3ʹ-UTR could bind to miR-125a-5p. MiR-125a-5p is a member of the miR-125 family and can regulate a variety of diseases. Currently, studies have found that miR-125a-5p can regulate proliferation of VSMCs [12]. However, the relationship between IRF1 and miR-125a-5p during VSMCs proliferation has not been shown. Furthermore, in immune thrombocytopenic purpura, MEG3 was involved in the regulation of immune imbalance through inhibiting miR-125a-5p [13], though the regulatory role of MEG3/miR-125a-5p in the proliferation of VSMCs has not been reported.

Here, we investigated the role of HDAC4 and MEG3/miR-125a-5p/IRF1 on VSMCs proliferation, aiming to discover new therapeutic targets for IH.

## Methods

### Cell culture and transfection

VSMCs were isolated from the thoracic aortas of healthy Sprague-Dawley (SD) rats by using enzymatic dissociation as described previously [6]. VSMCs were cultured in Dulbecco’s modified Eagle’s medium (DMEM) containing 10% fetal bovine serum (FBS, Invitrogen, USA), and 100 U/mL of penicillin-streptomycin. The medium was changed every 2–3 days. To induce the proliferation and migration of VSMCs, cells were treated with 20 ng/ml of platelet derived growth factor (PDGF)-BB (Shanghai Haoran Biological Technology Co., Ltd. China) for 24 h. Then, si-HDAC4, si-MEG3, si-IRF1, miR-125a-5p inhibitor and miR-125a-5p mimic (GenePharma, Shanghai, China) were transfected into VSMCs using Lipofectamine 2000 (Invitrogen) according to the manufacturer’s instruction. Twenty-four hours after transfection, VSMCs were harvested for qRT-PCR or western blot analysis. The sequences for all genes used in this study were shown in Supplementary Table 1.

### Quantitative real-time PCR (qRT-PCR)

Total RNA from cells was extracted using TRIzol reagent (Invitrogen), and the RNA purity was measured with an ultraviolet spectrophotometer. The mRNA expression of the target genes was quantified by using the SYBR Green PCR Master Mix with the ABI StepOnePlus Real-Time PCR system (Applied Biosystems, USA). GAPDH was used as the internal control for the expression of MEG3, and U6 was used as the internal control for the expression of miR-125a-5p. Data were analyzed by using comparative 2^−ΔΔCt^ method. The sequences of the primers used were shown in Supplementary Table 2.

### Western blot

The proteins from cells or tissues were extracted using RIPA buffer (Beyotime, Beijing, China) on the ice for 15 min and centrifuged at 12,000 rpm for 15 min. Then, the protein concentrations were determined by BCA Protein Assay kit (Pierce Biotechnology) according to the manufacturer’s instructions. Samples containing equal protein concentrations were separated by 12% SDS-PAGE and transferred onto PVDF membranes (Millipore, USA), which were then blocked with 5% skim milk for 1 h and incubated with the first primary antibody overnight at 4°C. Anti-IRF1 antibody, anti-HDAC4 antibody and anti-β-actin were used as the first primary antibody at 1:1000 dilutions. After primary antibody incubation, the membranes were incubated with the corresponding horseradish peroxidase-conjugated secondary antibody at room temperature for 1 h. The enhanced chemiluminescence system (ECL, Roche Molecular Biochemicals) was used to detect protein-antibody complexes. β-actin was served as a positive control to quantify the expression of related proteins.

### CCK-8 assay

VSMCs proliferation was measured using CCK-8 Assay Kit (Solarbio, USA) according to the manufacturer’s instructions. Brieﬂy, VSMCs were seeded into a 96-well culture plate (1 × 10^6^ cells/well) and cultured in DMEM medium containing 10% FBS for 24 h. Then, 10 μl of CCK-8 was added into each well. After 2 h incubation, the optical density (OD) values were measured at 450 nm using an enzyme immunoassay analyzer.

### Transwell assay

The migration of VSMCs was detected by Transwell assay. VSMCs were transfected with miR-125a-5p inhibitor or/and si-IRF1 for 24 h. Then, the cells were harvested and VSMCs suspensions (1 × 10^6^ cells) added to each well of the upper chamber of the Transwells, and the lower chamber was filled with 500 μL of DMEM containing 10% FBS. After 24 hours incubation, the cells on the upper surface were removed using a cotton swab, and the cells on the lower surface of the membrane were fixed with 4% paraformaldehyde and stained with 0.5% crystal violet for 10 min. The cells were counted in five random visual fields under a microscope.

### Chromatin immunoprecipitation (ChIP) assay

ChIP assay was performed as described previously [14,15]. The PDGF-BB (20 ng/ml) stimulated VSMCs were fixed with 1% formaldehyde for 10 min, then glycine was added for 5 min to terminate the fixation. After that, the cells were lysed and collected in EP tubes. Next, the DNA fragments were disrupted using ultrasound for 5 min, and centrifugated at 12,000 rpm/min for 10 min. The supernatant was collected, and anti-HDAC4 antibody and IgG antibody was added. After overnight incubation at 4°C, protein A/G was added to the complexes and incubated for 2 h. These were then washed, eluted, and removed cross‑linking. Finally, the bound DNA fragments were quantified by qPCR using SYBR Green Master Mix (Applied Biosystems).

### Double luciferase reporter assay

The 3ʹUTR of IRF1 was constructed such that it included the conserved binding sites for miR-125-5p and a mutant 3ʹUTR fragment of IRF1 was constructed with the mutations confined to the conserved binding sites for miR-125-5p. The fragments that included the 3ʹUTR regions (IRF1-WT) or mutant 3ʹUTR regions (IRF1-WT) were inserted into vector pcDNA containing a firefly luciferase reporter gene. VSMCs were co-transfected with reporter plasmid and miR-125a-5p mimic or miR-125a-5p inhibitor using Lipofectamine 2000 (Invitrogen) for 48h. Next, the cells were collected, and their luciferase activity was measured using dual Luciferase detection system (Promega) according to the manufacturer’s instructions.

### Vein graft model

Male SD rats (300–350 g, 10–12 weeks old) were purchased from Shanghai Bioray Laboratory Inc. The vein graft model was established as described previously [16]. Brieﬂy, an incision was made in the midline of the neck after the rats were anesthetized. Then, the left external jugular vein was harvested and stored in heparin-containing saline (50 IU· mL^−1^). The left carotid artery was removed from the surrounding connective tissues and ligated with two noninvasive blood vessel clamps to block the blood flow, and then cut in the middle. Each end of the carotid artery was passed through an outer tube (BD Biosciences, USA), everted, and fixed with 3–0 sutures. Next, the segment of external jugular vein was sleeved over the outer tubes and fixed with 3–0 sutures. Then, the noninvasive blood vessel clamps were removed, the blood ﬂow was re-established, and the skin incision was sutured. These vein graft rats were divided into miR-125a −5p inhibitor group (subcutaneous injection of LV-miR-125a −5p inhibitor, 108 pfu/mL) and control group (NC group, subcutaneous injection of LV-NC, 108 pfu/mL). After 4 weeks, the graft vein was collected and fixed in 4% paraformaldehyde at 4°C for 24 h, then embedded in paraffin, sectioned and stained with hematoxylin and eosin (HE). The neointimal thickness was measured using Digimizer 3.1.1.0 Image analysis software. The Care and Use of Laboratory Animals issued by the Chinese Association for Laboratory Animal Care approved all the procedures.

### Statistical analysis

SPSS 17.0 was used for the data analysis. The measurement data were presented as mean ± standard deviation (SD). Relative levels of MEG3 and miR-125a-5p in treated groups were compared to control group using Student’s *t*-test. Results of *in vitro* proliferation and migration experiments were compared using the one-way ANOVA test. *P *< 0.05 was considered statistically significant.

## Results

### The expression of HDAC4, MEG3, miR-125a-5p and IRF1 in VSMCs induced by PDGF-BB

**Figure 1.**
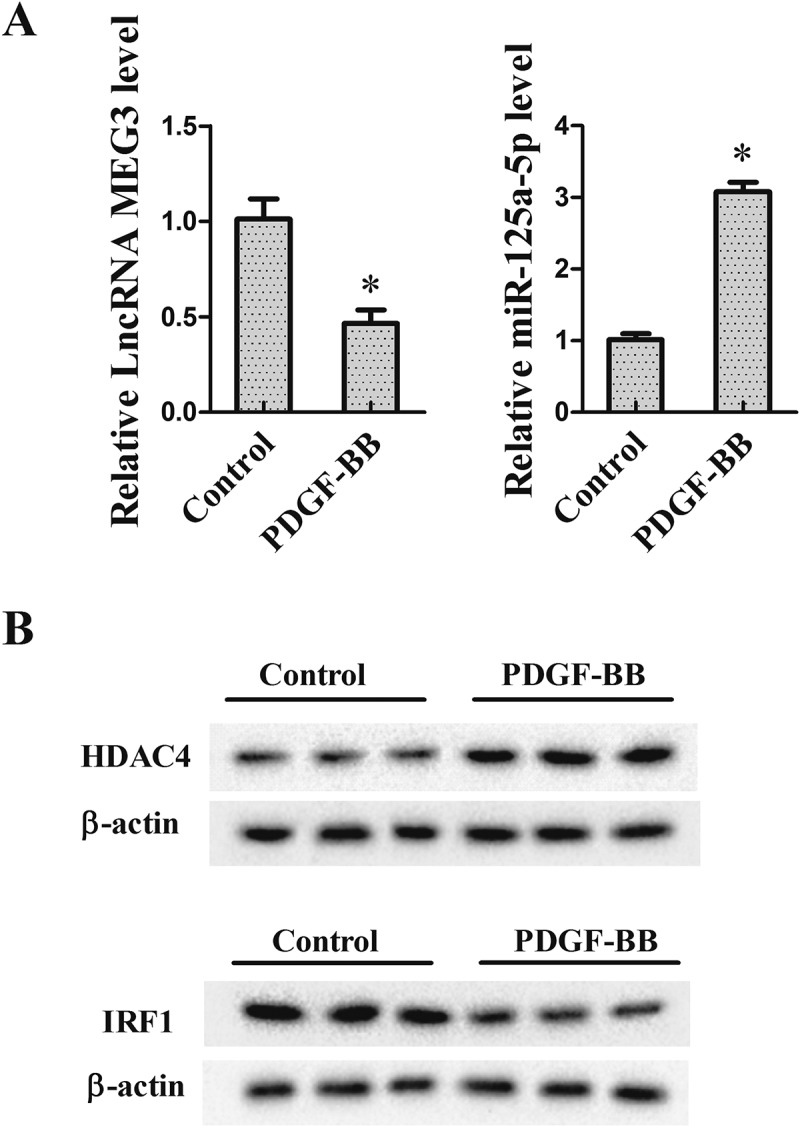
The expression of HDAC4, MEG3, miR-125a-5p and IRF1 in VSMCs induced by PDGF-BB. VSMCs were isolated from the thoracic aortas of health SD rats by using enzymatic dissociation, then cultured in DMEM medium containing 10% FBS. In the experiment, VSMCs were divided into control group and PDGF-BB group (cells were treated with 20 ng/ml PDGF-BB for 24 h). (a) The expression of MEG3 and miR-125a-5p was detected using qRT-PCR. (b) The protein expression of HDAC4 and IRF1 was measured by western blot. *P < 0.05, *vs* control group.

In the experiment, VSMCs were divided into control group and PDGF-BB group, wherein the cells in PDGF-BB group were treated with 20 ng/ml of PDGF-BB for 24 h to simulate the abnormal proliferation of VSMCs in IH. As shown in Figure 1(a), MEG3 expression was decreased in the PDGF-BB group compared to the control group, while miR-125a-5p expression was increased. Moreover, western blot results showed that HDAC4 expression was higher in the PDGF-BB group than in the control group, but IRF1 protein expression was lower (Figure 1(b)). The expressions of the four molecules in different time points (6, 12 and 24 hours after VSMC treated with PDGF-BB) were shown in Supplementary Figure 1.

### The interaction between MEG3 and HDAC4

**Figure 2.**
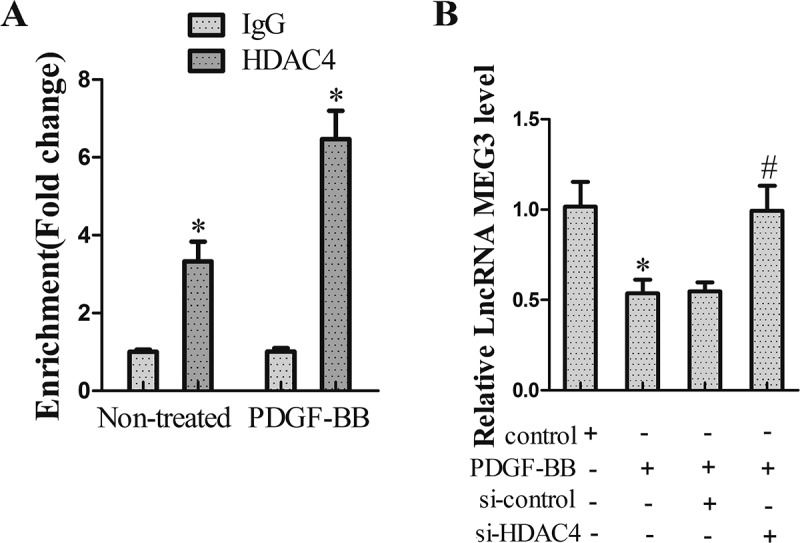
The interaction between MEG3 and HDAC4. (a) The relationship between MEG3 and HDAC4 was measured using ChIP assay. *P < 0.05, *vs* IgG. (b) VSMCs were divided into control group, PDGF-BB group, PDGF-BB+ si-control group and PDGF-BB+ si-HDAC4 group. qRT-PCR was used to determine MEG3 expression. *P < 0.05, *vs* control group; #*P *< 0.05, *vs* PDGF-BB+ si-control group.

To investigate whether HDAC4 could bind to the region of the MEG3 promoter, ChIP assay was performed. Results revealed that there was more binding of HDAC4 on MEG3 promoter in VSMCs induced by PDGF-BB (Figure 2(a)). Then, cell transfection was carried out to explore the regulatory effect. VSMCs were divided into control group, PDGF-BB group, PDGF-BB+ si-control group and PDGF-BB+ si-HDAC4 group. qRT-PCR results showed that the treatment of PDGF-BB reduced MEG3 expression, while HDAC4 interference could reverse the reduction (Figure 2(b)).

### HDAC4 regulated miR-125a-5p expression through MEG3

**Figure 3.**
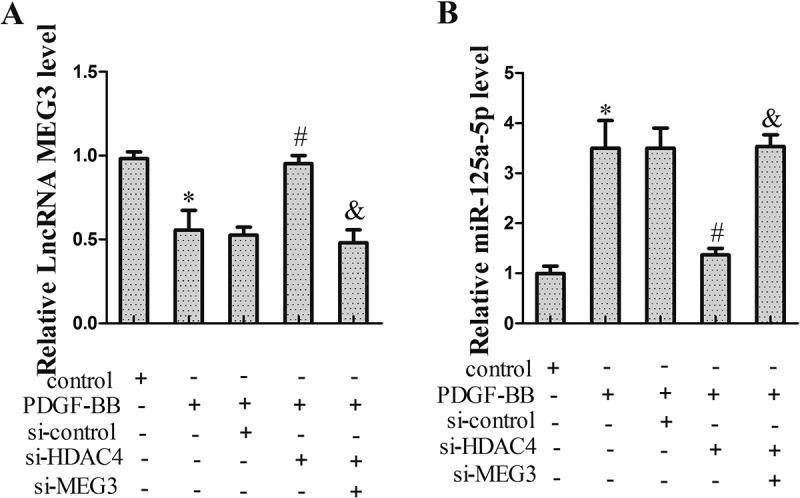
HDAC4 regulated miR-125a-5p expression through MEG3. VSMCs were divided into control group, PDGF-BB group, PDGF-BB+ si-control group, PDGF-BB+ si-HDAC4 group and PDGF-BB+ si-HDAC4+ si-MEG3 group. Twenty-four hours after transfection, VSMCs were harvested for qRT-PCR or western blot. (a) MEG3 expression. (b) miR-125a-5p expression. *P < 0.05, *vs* control group; #*P *< 0.05, *vs* PDGF-BB+ si-control group; &*P *< 0.05, *vs* PDGF-BB+ si-HDAC4 group.

In this experiment, we searched how HDAC4 regulated miR-125a-5p. After treatment with 20 ng/ml PDGF-BB for 24 h, VSMCs were transfected with si-HDAC4 and si-MEG3 for 24 h. As shown in Figure 3(a), MEG3 expression was up-regulated in VSMCs transfected with si-HDAC4, but si-MEG3 countered this augmentation (Figure 3(a)). Meanwhile, si-HDAC4 inhibited the expression of miR-125a-5p, though the suppression effect was abolished by si-MEG3 (Figure 3(b)).

### Interference of miR-125a-5p inhibited VSMCs proliferation

**Figure 4.**
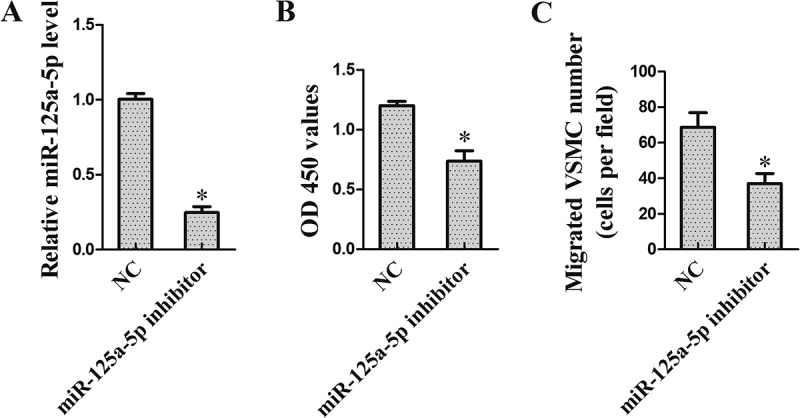
Interference of miR-125a-5p inhibited VSMCs proliferation. VSMCs were transfected with miR-125a-5p inhibitor. Twenty-four hours after transfection, VSMCs were harvested for the following research. (a) miR-125a-5p expression. (b) VSMCs proliferation was detected using CCK-8 assay. (c) VSMCs migration was tested using Transwell assay. *P < 0.05, *vs* NC.

To explore the role of miR-125a-5p on VSMCs proliferation, VSMCs were transfected with miR-125a-5p inhibitor for 24 h. qRT-PCR results showed that miR-125a-5p expression was decreased in VSMCs after transfected with miR-125a-5p inhibitor (Figure 4(a)). In addition, CCK-8 and Transwell assays showed that miR-125a-5p inhibitor could repress the proliferation (Figure 4(b)) and migration (Figure 4(c)) of VSMCs.

### miR-125-5p directly regulated IRF1

**Figure 5.**
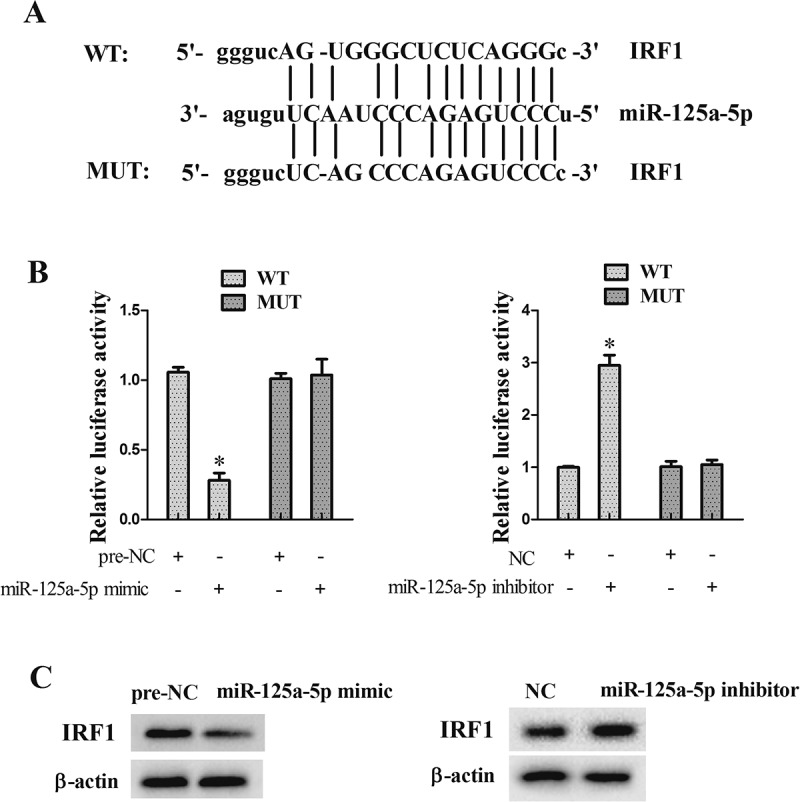
miR-125-5p directly regulated IRF1. (a) Software prediction (microRNA.org) showed that miR-125a-5p might combine with IRF1 3 ‘UTR. (b) Double luciferase reporter assay was used to determine whether miR-125-5p directly regulated IRF1 in VSMCs. (c) Overexpression of miR-125a-5p down-regulated IRF1 expression, while the interference of miR-125a-5p up-regulated IRF1 expression. *P < 0.05, *vs* NC or per-NC.

Prediction Software (microRNA.org) indicated that miR-125a-5p might combine with IRF1 3ʹUTR (Figure 5(a)). To determine whether miR-125-5p directly regulated IRF1 in VSMCs, Double luciferase reporter assay was performed. Results showed that the luciferase activity of IRF1-WT was reduced in VSMCs transfected with miR-125a-5p mimic and enhanced in VSMCs transfected with miR-125a-5p inhibitor (Figure 5(b)). In addition, western blot data revealed that overexpression of miR-125a-5p down-regulated IRF1 expression, while miR-125a-5p interference up-regulated IRF1 expression (Figure 5(c)).

### miR-125a-5p regulated VSMCs proliferation through IRF1

**Figure 6.**
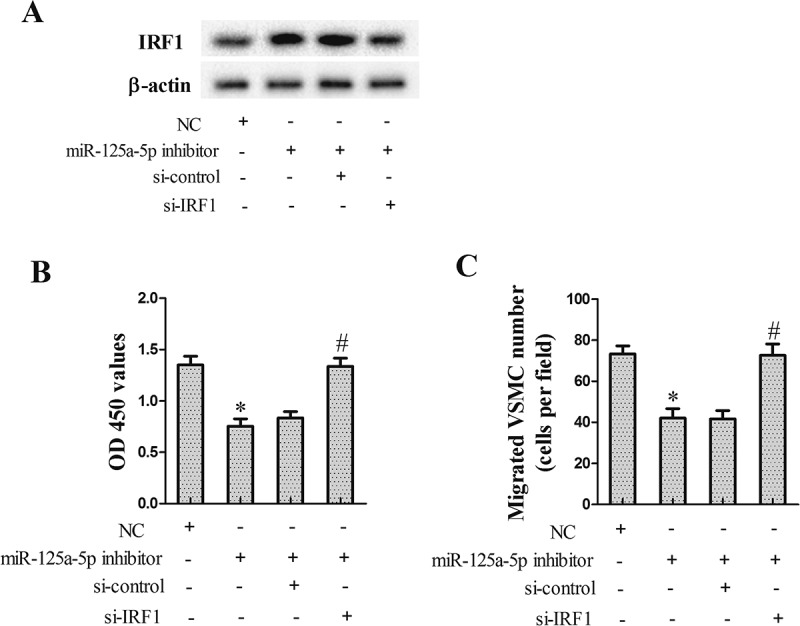
miR-125a-5p regulated VSMCs proliferation through IRF1. VSMCs were divided into NC group, miR-125a-5p inhibitor group, miR-125a-5p inhibitor+ si-control group, and miR-125a-5p inhibitor+ si-IRF1 group. Twenty-four hours after transfection, VSMCs were harvested for the following research. (a) IRF1 protein repression. (b) VSMCs proliferation. (c) VSMCs migration. *P < 0.05, *vs* NC group; #*P *< 0.05, *vs* miR-125a-5p inhibitor+ si-control group.

In this experiment, we tested how miR-125a-5p/si-IRF1 regulated VSMCs proliferation. After treatment with 20 ng/ml PDGF-BB for 24 h, VSMCs were transfected with miR-125a-5p inhibitor and si-IRF1 for 24 h. Results showed that miR-125a-5p inhibitor could promote IRF1 protein expression (Figure 6(a)), and inhibit the proliferation (Figure 6(b)) and migration (Figure 6(c)) of VSMCs, though si-IRF1 reversed these effects.

### The effect of miR-125a-5p on neointimal formation in vivo

**Figure 7.**
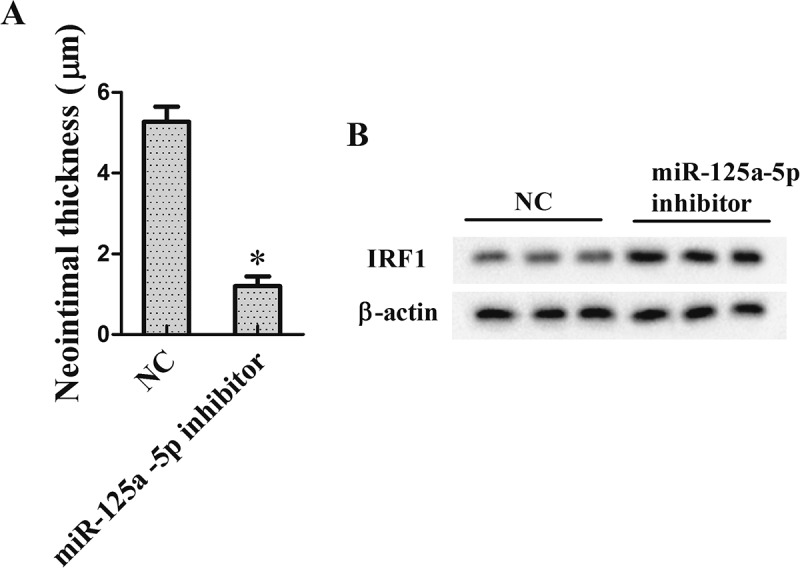
The effect of miR-125a-5p on neointimal formation *in vivo*. To verify the effect of miR-125a-5p on neointimal formation, we performed vein grafts in rats. Rats were divided into miR-125a −5p inhibitor group (subcutaneous injection of LV-miR-125a −5p inhibitor, 108 pfu/mL) and control group (NC group, subcutaneous injection of LV-NC, 108 pfu/mL). Four weeks after operation, the graft vein was collected for the following research. (a) Neointimal thickness. (b) IRF1 protein repression. *P < 0.05, *vs* NC.

To verify the effect of miR-125a-5p on neointimal formation, we performed vein grafts in rats. Rats were divided into miR-125a-5p inhibitor group (subcutaneous injection of LV-miR-125a-5p inhibitor, 108 pfu/mL) and control group (NC group, subcutaneous injection of LV-NC, 108 pfu/mL). Four weeks after operation, the graft vein was collected for the following research. As shown in Figure 7(a), miR-125a-5p inhibitor reduced the neointimal thickness, which indicated an inhibition effect of miR-125a-5p inhibitor on IH. Additionally, western blot results showed that IRF1 expression in grafted tissue was increased in the presence of the miR-125a-5p inhibitor (Figure 7(b)).

## Discussion

The abnormal proliferation of VSMCs is an important cause of IH. Therefore, finding potential targets that would interfere with VSMCs proliferation has great significance in the alleviation and treatment of IH. HDAC4 has been considered as a critical regulator of cell proliferation in various cell types, such as satellite cells [17], gastric cancer cells [18], and neural progenitors [5]. In addition, several studies have demonstrated a regulatory effect of HDAC4 on VSMCs. Usui et al. [19] showed that HDAC4 small interfering RNA inhibited PDGF-BB-induced VSMCs proliferation and migration. Ginnan et al. [6] revealed that overexpression of HDAC4 promoted VSMCs proliferation. However, the mechanisms of HDAC4 on VSMCs proliferation have not been thoroughly investigated. In the present study, we found an increased expression of HDAC4 in PDGF-BB-induced VSMCs, which was consistent with a previous study [19]. To elucidate the underlying mechanism of HDAC4 on VSMCs proliferation, we examined whether HDAC4 regulated the expression of MEG3.

MEG3, an imprinted gene, is located at chromosome 14q32.3 in the human genome. Low MEG3 expression has been found in various diseases, including cancers, osteoarthritis [20], and coronary artery disease [21]. Sun et al. [22] revealed that MEG3 knockdown could promote the proliferation of human pulmonary artery smooth muscle cells, and Liu et al. [8] reported that MEG3 overexpression could inhibit VSMC proliferation. In the current study, we found that the expression of MEG3 was decreased in PDGF-BB-induced VSMCs, and the interference of HDAC4 could counter this repressive effect of PDGF-BB on MEG3 expression, which indicated that HDAC4 might participate in VSMCs proliferation by regulating MEG3. Further research confirmed this using cell transfection experiments that showed HDAC4 knockdown inhibited the proliferation and migration of VSMCs via upregulating MEG3 and downregulating miR-125a-5p.

MiRNAs are a type of non-coding small RNAs that are usually 18–25 nucleotides in length and can serve important roles in various cellular biology processes. It is reported that some miRNAs can regulate VSMCs proliferation. For instance, Chen et al. [23] demonstrated that miR-612 expression was decreased in PDGF-BB-induced VSMCs, and overexpression of miR-612 significantly inhibited PDGF-BB-induced proliferation of VSMCs. Ham et al. [24] reported that increased miR-9 expression inhibited VSMCs proliferation *in vitro*. In our study, miR-125a-5p expression was increased in PDGF-BB-induced VSMCs, and inhibition of miR-125a-5p could attenuate the proliferation and migration of VSMCs induced by PDGF-BB. This is the first report of the regulatory relationship between miR-125a-5p and VSMCs. Moreover, we found that miR-125a-5p affected the proliferation and migration of VSMCs by directly regulating IRF1.

IRF1 is closely related to the biological processes of VSMCs. Studies have showed that IRF1 can participate in the regulation of VSMCs proliferation [11] and apoptosis [25]. These results are in line with our findings. To verify the mechanism of these molecules, we performed animal experiments. Our results demonstrated that inhibition of miR-125a-5p could alleviate IH by increasing IRF1 expression.

In summary, HDAC4 interference could inhibit PDGF-BB-induced VSMCs proliferation via regulating the MEG3/miR-125a-5p/IRF1 axis (the flow chart was shown in Supplementary Figure 2). This finding provided new potential therapeutic targets for the treatment of IH, and a new research field for the treatment of vascular proliferative disorders.
