# Supplementary figures and images for: Interfering histone deacetylase 4 inhibits the proliferation of vascular smooth muscle cells via regulating MEG3/miR-125a-5p/IRF1
Source: Cell Adh Migr. 2018 Aug 29;13(1):41–9. doi: 10.1080/19336918.2018.1506653 (PMC6527374; doi:10.1080/19336918.2018.1506653)

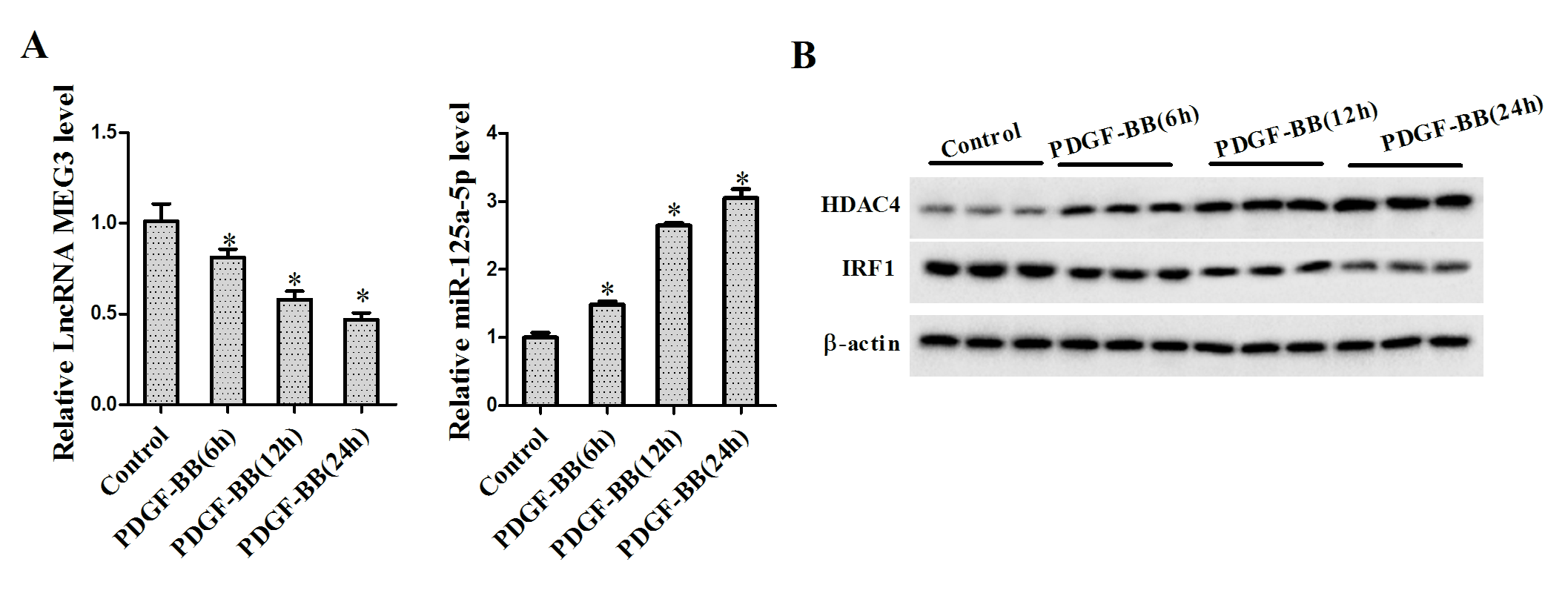

Supplement: Supplemental Material [file kcam-13-01-1506653-s001.zip › Supplementary Figure 1.tif]

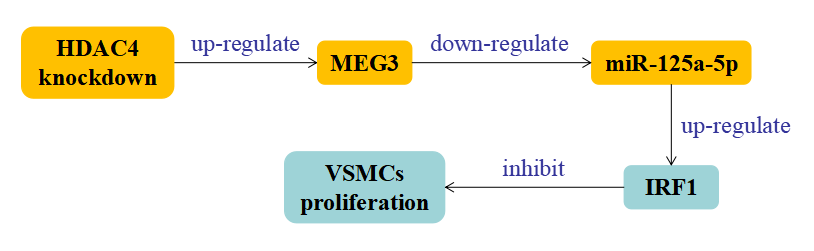

Supplement: Supplemental Material [file kcam-13-01-1506653-s001.zip › Supplementary Figure 2.tif]
