# Supplementary material for: Interfering histone deacetylase 4 inhibits the proliferation of vascular smooth muscle cells via regulating MEG3/miR-125a-5p/IRF1
Source: Cell Adh Migr. 2018 Aug 29;13(1):41–9. doi: 10.1080/19336918.2018.1506653 (PMC6527374; doi:10.1080/19336918.2018.1506653)
Supplement: Supplemental Material [file kcam-13-01-1506653-s001.zip › Supplementary Table 1.doc]

**Supplementary Table 1** Primers for transfection experiment

| **Gene** | **Sequences** |
| --- | --- |
| si-MEG3 | 5’-GACUUAAACCAAUGCCCUA-3’ |
| si-HDAC4 | 5’-GCAGAUCUGUGUUUUGAAA-3’ |
| si-IRF1 | 5’-GAAGATAGCCGAAGACCTTTT-3’ |
| miR-125a-5p inhibitor | 5’-UCACAAGUUAGGGUCUCAGGGA-3’ |
| miR-125a-5p mimic | 5’-UCCCUGAGACCCUUUAACCUGUGA-3’ |
