# Supplementary material for: Interfering histone deacetylase 4 inhibits the proliferation of vascular smooth muscle cells via regulating MEG3/miR-125a-5p/IRF1
Source: Cell Adh Migr. 2018 Aug 29;13(1):41–9. doi: 10.1080/19336918.2018.1506653 (PMC6527374; doi:10.1080/19336918.2018.1506653)
Supplement: Supplemental Material [file kcam-13-01-1506653-s001.zip › Supplementary Table 2.doc]

**Supplementary Table 2** Primers for qRT-PCR

| **Gene** | **Sequences** |
| --- | --- |
| MEG3 | Forward: 5’-CTGCCCATCTACACCTCACG-3’ |
|  | Reward: 5’-CTCTCCGCCGTCTGCGCTAGGGGCT-3’ |
| GAPDH | Forward: 5’-GTCAACGGATTTGGTCTGTATT-3’ |
|  | Reward: 5’-AGTCTTCTGGGTGGCAGTGAT-3’ |
| miR-125a-5p | Forward: 5’-GGGTCCGAGGTATTCGCACT-3’ |
|  | Reward: 5’-TCCCTGAGACCCTTTAACCT-3’ |
| U6 | Forward: 5’-CTCGCTTCGGCAGCACA-3’ |
|  | Reward: 5’-AACGCTTCACGAATTTGCGT-3’ |
